# TGF-β-Induced Endothelial to Mesenchymal Transition in Disease and Tissue Engineering

**DOI:** 10.3389/fcell.2020.00260

**Published:** 2020-04-21

**Authors:** Jin Ma, Gonzalo Sanchez-Duffhues, Marie-José Goumans, Peter ten Dijke

**Affiliations:** ^1^Department of Cell and Chemical Biology, Leiden University Medical Center, Leiden, Netherlands; ^2^Oncode Institute, Leiden University Medical Center, Leiden, Netherlands

**Keywords:** cancer-associated fibroblast, cardiovascular disease, EndMT, fibrosis, signal transduction, Smad, TGF-β, tissue regeneration

## Abstract

Endothelial to mesenchymal transition (EndMT) is a complex biological process that gives rise to cells with multipotent potential. EndMT is essential for the formation of the cardiovascular system during embryonic development. Emerging results link EndMT to the postnatal onset and progression of fibrotic diseases and cancer. Moreover, recent reports have emphasized the potential for EndMT in tissue engineering and regenerative applications by regulating the differentiation status of cells. Transforming growth factor β (TGF-β) engages in many important physiological processes and is a potent inducer of EndMT. In this review, we first summarize the mechanisms of the TGF-β signaling pathway as it relates to EndMT. Thereafter, we discuss the pivotal role of TGF-β-induced EndMT in the development of cardiovascular diseases, fibrosis, and cancer, as well as the potential application of TGF-β-induced EndMT in tissue engineering.

## Introduction

The cardiovascular system has the supportive role of supplying oxygen and nutrition to the whole body and simultaneously removes toxic waste products from tissues and organs through an extensive and intricated network of blood vessels. The inner surface of blood vessels consists of a monolayer of endothelial cells (ECs). These ECs, which may be supported by mural cells [i.e., pericytes and vascular smooth muscle cells (SMCs)], regulate the interchange between the luminal blood and the outer tissues (Pober and Sessa, 2007). During the development of the embryonic heart, a specific group of ECs lining the atrioventricular (AV) canal dedifferentiate into mesenchymal cells and migrate into the underlying extracellular matrix (ECM) to form the AV cushion (Markwald et al., 1975). This process of phenotypic switching of cardiac ECs was defined as endothelial to mesenchymal transition (EndMT) and thought to be regulated in part by the paracrine action of ligands secreted by the myocardium. Much of the mechanistic knowledge regarding EndMT has originated through studies focused on epithelial to mesenchymal transition (EMT). EMT is an evolutionarily conserved developmental process, induced by cytokines, mechanical forces, and metabolic factors (Saito, 2013; Wesseling et al., 2018), that has been shown to play a role in tumorigenesis and other pathophysiological processes (Heerboth et al., 2015; Pastushenko and Blanpain, 2018).

Notably, transforming growth factor β (TGF-β), a multifunctional cytokine secreted by the myocardium (among other tissues) with pleiotropic physiological roles, is one of the best studied EndMT (and EMT) inducers (Yoshimatsu and Watabe, 2011; Moustakas and Heldin, 2016). When ECs undergo EndMT, their tight cell–cell junctions are disrupted, causing ECs to lose their cobblestone-like and well-structured appearance, reorganize their cytoskeleton and turn into spindle-shaped, fibroblast-like cells. During this transitional process, the expression of cell–cell adhesion proteins, such as vascular endothelial (VE)-cadherin, platelet/EC adhesion molecule-1 (CD31/PECAM-1), tyrosine kinase with immunoglobulin-like and epidermal growth factor (EGF)-like domains 1 (TIE1), TIE2, and von Willebrand factor (vWF), are diminished, while mesenchyme-specific factors, including N-cadherin, α-smooth muscle actin (α-SMA), smooth muscle protein 22α (SM22α), vimentin, fibronectin, and fibroblast-specific protein-1 (FSP-1), are upregulated. These endothelial-derived mesenchymal cells gain stem cell properties as they can differentiate into different mesodermal cell types under certain conditions. Like EMT, EndMT is a gradual, reversible, and dynamic process. It is therefore difficult to capture in fixed biopsies; the presence of cells that express different levels of both endothelial and mesenchymal markers is suggestive that EndMT does occur. Partial EndMT is considered part of physiological angiogenesis (Welch-Reardon et al., 2015). ECs that have undergone partial EndMT were identified in the mouse heart (CD31/PECAM-1 and FSP-1) during the progression of cardiac fibrosis (Zeisberg et al., 2007b) as well as in the mouse brain (CD31/PECAM-1 and N-cadherin) in cerebral cavernous malformation (CCM) (Maddaluno et al., 2013).

In recent decades, the contribution of EndMT to human disease has been demonstrated in an increasing number of pathologies, including cardiovascular and fibrotic diseases and cancer (Medici et al., 2010; Souilhol et al., 2018; Platel et al., 2019). Increased TGF-β signaling has been suggested as a common underlying mechanism in almost every EndMT-associated disorder. Therefore, blocking TGF-β signaling might be a promising therapy for EndMT-related diseases. In contrast, because EndMT-derived mesenchymal multipotent cells can be used to generate various cell types within the mesodermal lineage, researchers have just begun to explore the potential of EndMT in tissue engineering, by recapitulating the EndMT process that occurs during embryogenesis and in organ development (Susienka and Medici, 2013). In this review, we summarize the mechanisms of TGF-β signaling and its role in driving EndMT. Furthermore, we discuss the role of EndMT in cardiovascular diseases, fibrosis, and cancer, as well as the potential applications of EndMT in tissue engineering.

## TGF-β Signaling

### Ligands

TGF-β signal transduction is involved in regulating a large number of cellular functions, including proliferation, migration and differentiation, and essential biological processes, such as embryonic development, the immune response, wound healing, angiogenesis, and cancer (Batlle and Massagué, 2019; Derynck and Budi, 2019). Since the discovery of TGF-β1 in the early 1980s due to its ability to induce the growth of normal rat kidney cells in soft agar, 33 human genes encoding polypeptide members belonging to the TGF-β family have been identified and characterized (Morikawa et al., 2016). TGF-β family members can be divided into subfamilies according to their sequences and functional similarities: TGF-βs, activins and nodal, bone morphogenetic proteins (BMPs), growth differentiation factors (GDFs), and anti-Müllerian hormone (AMH). Whereas TGF-βs were initially associated with the stimulation and inhibition of cell proliferation, activins (and their antagonists, termed inhibins) were first identified by their activity in the gonads (Xia and Schneyer, 2009). BMPs were discovered as molecules with the potential to induce ectopic cartilage and bone formation in rodents (Urist et al., 1982). These early discoveries have been followed by multiple studies that have unveiled the broad roles of each TGF-β family member in human (patho)physiology.

In response to extracellular stimuli (i.e., inflammation and hypoxia), TGF-βs are transcribed and secreted by cells in an inactive dimeric form. TGF-βs are inactive due to the non-covalent interaction between the amino-terminal pro-peptide sequence, known as latency-associated peptide (LAP), and the carboxy-terminal of the mature TGF-β peptide. When specific enzymes are activated, such as serine protease, plasmin and furin, the pro-peptide is cleaved thereby releasing TGF-β in a mature and active form. TGF-β family members may also be sequestered by binding to extracellular matrix (ECM) proteins or shielded from receptor binding by interacting with soluble antagonists. Together, these mechanisms carefully regulate TGF-β family member bioavailability (Brazil et al., 2015; Robertson and Rifkin, 2016).

### Receptors

TGF-β family members trigger biological processes by inducing the formation of cell surface receptor complexes bearing intrinsic serine/threonine kinase activity. Seven human type I receptors [activin receptor-like kinases (ALKs) 1–7] and five human TGF-β family type II receptors, i.e., activin type II A and B receptors (ActRIIA and ActRIIB), BMP type II receptor (BMPRII), TGF-β type II receptor (TβRII), and AMH type II receptor (AMHRII), have been identified. In the case of TGF-βs, their oligomeric receptor complexes comprise the type I (TβRI) and type II (TβRII) receptors (Cheifetz et al., 1990; Lin et al., 1992). Binding of TGF-β to TβRII promotes the recruitment of TβRI (also termed ALK5). While both TβRI and TβRII have intracellular kinase domains, only the type I receptor contains a glycine–serine-rich domain (GS domain) at its juxtamembrane region. Specific serine and threonine residues in the GS domain are phosphorylated by TβRII kinase, resulting in TβRI activation (Wrana et al., 1994). In addition to TβRI and TβRII, there are a number of TGF-β coreceptors (including Endoglin, TβRIII (also termed betaglycan) and Cripto) that contain a short (or lack an) intercellular domain without kinase activity and fine-tune the interaction between extracellular ligands and membrane receptor complexes, thereby modulating cellular responses to TGF-β stimulation (Nickel et al., 2017). While there are differences in how TGF-β family members engage their cell surface receptors, the notion that ligand-induced receptor complex formation mediates type I phosphorylation and activation by type II kinase is common to all TGF-β family members and their signaling receptors.

### Intracellular Signaling

**FIGURE 1:**
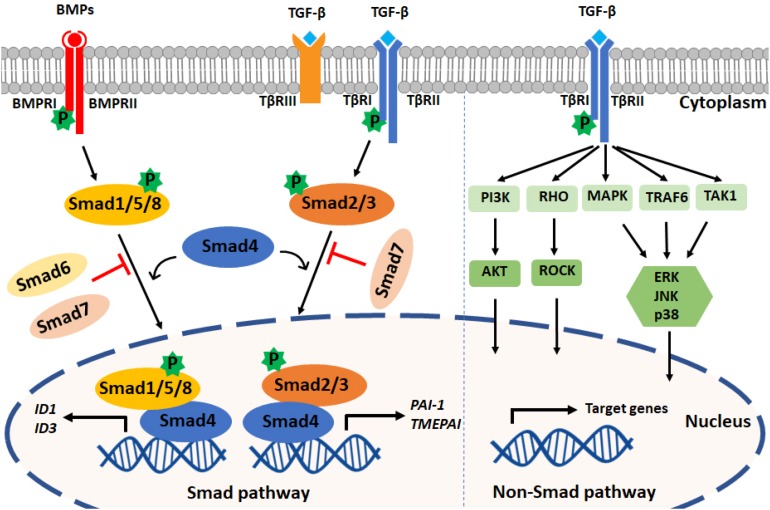
TGF-β family signaling pathways. **Left panel:** TGF-β family ligands signal via type I and type II receptors on the cell surface. Upon ligand engagement, the type II kinases transphosphorylate the type I receptors, which are then activated. TGF-βs and BMPs are shown as an example; TGF-βs bind TβRI and TβRII, and BMPs bind BMPRI and BMPRII. TβRIII (also termed Betaglycan) is a coreceptor that facilitates interaction with TβRI and TβRII. TGF-βs induce the phosphorylation of Smad2/3, and BMPs mediate Smad1/5/8 phosphorylation. By forming complexes with Smad4, phosphorylated Smad2/3 and Smad1/5/8 translocate into the nucleus to regulate target gene expression. *PAI1* and *TMEPAI* are typical target genes induced downstream of Smad3 phosphorylation, and *Id1* and *Id3* are induced after Smad1 and Smad5 activation. Inhibitory Smads (i.e., Smad6 and Smad7) can antagonize the action of signal-transducing R-Smads and Smad4. **Right panel:** TGF-β family members can also activate PI3K, RHO, MAPK, TRAF6, and TAK1 through non-Smad pathways. TGF-β is shown, but these non-Smad signaling pathways can also be activated by BMPs and other family members.

Upon type I receptor activation, the signal is transduced from the cell membrane into the nucleus by phosphorylation of a specific subset of intracellular transcriptional effector proteins, termed mothers against decapentaplegic and Sma homologs or Smads (Derynck et al., 1996; Figure 1). Smad proteins can be classified into three groups: (1) receptor-associated Smads (R-Smads, Smad1/2/3/5/8), (2) common Smad (i.e., co-Smad, also known as Smad4 in vertebrates), and (3) inhibitory Smads (I-Smads, Smad6/7) (Hill, 2016). By using different receptor complexes, ligands of the TGF-β family induce the phosphorylation and activation of specific R-Smads. For example, TGF-βs (via TβRI/ALK5) and activins (via ALK4/7) induce the phosphorylation of Smad2 and Smad3, whereas BMPs, upon activating ALK1/2/3/6, signal via Smad1/5/8. Activated R-Smads then associate with the co-Smad, i.e., Smad4, to form heteromeric complexes. These complexes can translocate into the nucleus, where they regulate specific gene transcriptional responses (Shi and Massagué, 2003; Derynck and Budi, 2019). In general, while Smad1/5/8 promote the induction of genes involved in proliferation and osteogenic differentiation (i.e., *Id-1/3* and *Runx2*), Smad2/3 induce the expression of pro-fibrotic genes (i.e., *Serpine-1* and *Collagen tissue growth factor*). Smad6 and Smad7 antagonize TGF-β family-induced signal transduction by inhibiting the stability or function of the activated receptors or by interacting with Smad4 to prevent the heteromeric complex formation of activated R-Smads and Smad4 (Itoh and ten Dijke, 2007).

In addition to the so-called canonical Smad pathway described above, TGF-β family members can signal via non-Smad pathways, such as the extracellular signal-regulated kinase (Erk) MAP kinase (MAPK), Rho-like GTPase, phosphatidylinositol-3-kinase (PI3K)/AKT, p38 MAPK, Jun amino-terminal kinase (JNK), ubiquitin ligase tumor necrosis factor (TNF)-receptor associated factor 6 (TRAF6), and TGF-β activated kinase 1 (TAK1) pathways. The non-Smad signaling pathways act in a context-dependent manner and will fine-tune cell-specific biological processes (Zhang, 2017). Notably, the Smad and non-Smad pathways engage in crosstalk, e.g., ERK MAPK, which can be activated through the non-Smad pathway, is able to engage in crosstalk with the Smad pathway to regulate Smad2/3 phosphorylation (Hayashida et al., 2003; Zhang, 2009).

## TGF-β-Induced EndMT

### TGF-β Family Members in EndMT

**FIGURE 2:**
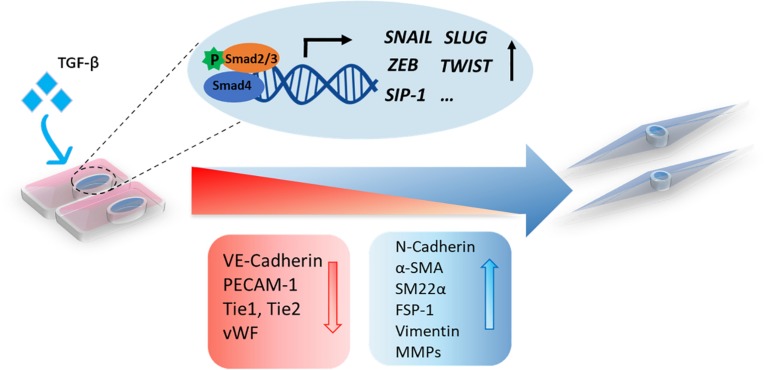
A schematic representation of TGF-β-induced EndMT. The activation of TGF-β signaling leads to the accumulation of nuclear Smad transcription factor complexes. These complexes can induce the expression of transcription factors (*SNAIL*, *SLUG*, *ZEB*, *TWIST*, and *SIP-1*) and trigger EndMT in which cell morphological changes occur, including a switch from the cobblestone-like endothelial morphology to a spindle-like fibroblast morphology. Upon EndMT, endothelial cells lose polarity, and the expression of endothelial markers, such as VE-cadherin, PECAM-1, Tie1, Tie2, and Vwf, is decreased, while the expression of mesenchymal markers, including N-cadherin, α-SMA, SM22α, FSP-1, vimentin, and MMPs, is increased.

EndMT is a process of pivotal importance for proper cardiac cushion formation during embryonic development (Markwald et al., 1975; Eisenberg and Markwald, 1995; Brown et al., 1999). Similar to EMT, a variety of autocrine and paracrine signaling molecules can drive EndMT, including TGF-β, Wnt/β-catenin Notch, and inflammatory cytokines (Watabe et al., 2003; Kokudo et al., 2008; Pérez et al., 2017; Wang et al., 2018; Zhong et al., 2018; Sánchez-Duffhues et al., 2019a). In recent years, valuable insights regarding the role of TGF-β family members in controlling the dynamic EndMT process have been obtained (Figure 2). All three mammalian isoforms of TGF-β (TGF-β1, TGF-β2, and TGF-β3) can induce EndMT, although different isoform- and species-specific functions have been reported (Goumans et al., 2008; Pardali et al., 2017). Recently, Sabbineni et al. (2018) showed that in human dermal microvascular ECs (HMECs) TGF-β2 is more potent than TGF-β1 or TGF-β3 in inducing the expression of the mesenchymal transcription factors Snail and FoxC2. Treatment with TGF-β1 and TGF-β3 induced the expression of TGF-β2, suggesting that they can act in an indirect manner. Furthermore, TGF-β2-induced EndMT has been reported to increase the pool of cancer-associated fibroblasts (CAFs) in colon cancer (Wawro et al., 2019). The function of TGF-β signaling in regulating EndMT *in vivo* has been interrogated in part by investigating different transgenic and knockout animal models. Both TGF-β2 and TGF-β3 were shown to be required for the EndMT process involved in the formation of AV cushions in chick embryos (Camenisch et al., 2002). By histological examination of cushion morphology in E14.5-specific TGF-β deficient mouse embryos, no obvious valvular defects were observed in *Tgfb1*- or *Tgfb3*-knockout mice. *Tgfb2* deficient mice, however, demonstrated multiple defects in AV cushion formation. This is line with the observation that only TGF-β2 is strongly expressed in the cushion myocardium and invading mesenchymal cells in mice (Brown et al., 1999; Azhar et al., 2009). Furthermore, Jiao et al. (2006) used the Cre/loxp system to specifically inactivate the TβRII in mice. They showed that inactivation of this receptor in either the myocardium or the endothelium of mouse embryos did not prevent EndMT and AV cushion formation, suggesting that other TGF-β family ligands compensate for this pathway (Jiao et al., 2006). While BMPs were found to induce EndMT *ex vivo* and *in vitro*, the specific deletion of different BMPs in mice did not unveil their functions in early cardiac differentiation due to the early lethality of the loss of specific BMPs or functional redundancy. While BMP5- or BMP7-deficient mice survived, without obvious cardiac abnormalities (Kingsley et al., 1992; Dudley and Robertson, 1997), the BMP5/7 double knockout mouse did show defects in AV cushion formation (Solloway and Robertson, 1999). BMP6-deficient mice did not show any cardiac abnormalities, although BMP6/BMP7 double-knockout mice did have cardiac defects (Solloway et al., 1998; Kim et al., 2001). BMP2 plays a vital role in modulating AV canal morphogenesis, as mice with BMP2 specifically inactivated in AV myocardium showed abnormal AV canal morphology at 9.5 days post coitum (dpc) and pericardial effusion and growth retardation at 10.5 dpc (Ma et al., 2005). ALK2 or ALK3 deficiency within the endothelium in mice resulted in AV canal defects, indicating that these two BMP type I receptors are important in inducing EndMT for endocardial cushion formation (Wang et al., 2005; Kaneko et al., 2008). Medici et al. (2010) showed that both TGF-β2 and BMP4 induce EndMT in human umbilical vein ECs (HUVECs) and human cutaneous microvascular ECs (HCMECs) in an ALK2- and TβRI-dependent manner. In summary, both the TGF-β and BMP signaling pathways have pivotal functions in EndMT.

### Transcription Factors Involved in TGF-β-Induced EndMT

TGF-β family members mediate EndMT via Smad or non-Smad signaling propagated by inducing the expression of specific transcription factors, such as Snail, Slug, Twist, ZEB1, SIP-1/ZEB2, and LEF-1 (Yoshimatsu and Watabe, 2011). The Snail family of transcription repressors, including Snail (*SNAI1*) and Slug (*SNAI2*), are the most studied downstream EndMT effectors induced by TGF-β (Kokudo et al., 2008; Medici et al., 2011). Snail family members are proteins containing four to six C2H2-type zinc finger motifs in their carboxy-terminal domain that bind a specific DNA region (E-box) (Nieto, 2002). Snail represses the expression of EC cell–cell adhesion molecules by binding to the promotor of *CDH5* (encoding for VE-cadherin) or *PECAM1* (encoding for CD31) and inducing the expression of mesenchymal markers such as *ACTA2* (encoding for α-SMA) (Kokudo et al., 2008; Cheng et al., 2013). Snail has a higher apparent DNA-binding affinity than Slug, which can result in more potent inhibition of endothelial specific target genes (Bolós et al., 2016). Kokudo et al. (2008) showed that Snail is essential for TGF-β2-induced EndMT in mouse embryonic stem cell-derived ECs (MESECs). Snail expression was upregulated by TGF-β2, whereas Snail knockdown abrogated TGF-β2-induced EndMT in these cells. The transcription factor Forkhead Box M1 (Foxm1), which can be induced by TGF-β, was found to drive EndMT by binding to Snail and enhancing its activity (Song et al., 2019). Twist can be transcriptionally activated in a signal transducer and activator of transcription (STAT)3 dependent manner by the recruitment of the transcriptional modulator megakaryoblastic leukemia (MKL)1 to its promotor region. Depletion of MKL1 or treatment with a Twist small molecule inhibitor attenuated TGF-β-induced EndMT in human vascular ECs (HVEC) and inhibited liver fibrosis in mice (Li et al., 2019). In addition, basic helix–loop–helix (bHLH) transcription factors, such as E2A (including E12 and E47) and ID (DNA-binding protein inhibitor), are master regulators of EMT (Lamouille et al., 2014). ID proteins bind E2A to form heterodimers and thereby regulate E2A activity (Slattery et al., 2008). The E2A protein contributes to EMT by regulating the expression of target genes, such as *ACTA2* (α-SMA) and *CDH1* (E-cadherin). Due to the similarity between the EMT and EndMT processes, the bHLH proteins might also play an important role in regulating EndMT.

### Interplay With Other Signaling Pathways That Mediate or Regulate EndMT

In addition to the Smad/non-Smad signaling pathway, TGF-β interacts with other signaling pathways that mediate and/or regulate EndMT, such as the Notch (Fu et al., 2009), fibroblast growth factor (FGF) (Chen and Simons, 2018), Wnt, and Sonic Hedgehog pathways (Horn et al., 2012). As such, Notch signaling is critical for heart formation during embryonic development (MacGrogan et al., 2018). TGF-β and Notch signaling cooperate to induce the expression of Snail, thereby downregulating the expression of VE-cadherin and promoting EndMT (Fu et al., 2009). In contrast, Patel et al. (2018) demonstrated that EC specific deletion of Notch signaling resulted in enhancement of EndMT since more CD31^–^FSP^+^ cells were detectable in skin wounds of endothelial specific transcription factor Rbpj-deficient mice. Interestingly, TGF-β1 expression was found to be increased in these CD34^–^/FSP-1^+^ wound ECs, which suggests that TGF-β is the main driver of EndMT in mice deficient for endothelial specific Notch signaling (Patel et al., 2018).

Several studies indicate that microRNAs (miRNAs) are regulated in response to TGF-β-induced EndMT. For example, Ghosh et al. (2012) reported that several miRNAs are regulated during TGF-β2-induced EndMT in mouse cardiac ECs (MCECs). After promoting EndMT by stimulating MCECs with TGF-β2 for 7 days, miR-125b, Let7C, Let-7g, miR-21, miR-30b, and miR-195 were upregulated while miR-122a, miR-127, miR-196, and miR-375 were downregulated (Ghosh et al., 2012). Correia et al. (2016) found that miR-20a is decreased during TGF-β1-induced EndMT. miR-20a regulates the expression levels of the TGF-β receptors TβRI and TβRII. FGF2 was found to induce miR-20a and antagonize TGF-β1-induced EndMT (Correia et al., 2016).

Fibroblast growth factor (FGF) is known to inhibit TβRI expression (Fafeur et al., 1990). An increasing number of studies have shown that FGF and TGF-β crosstalk in more complex ways. Endothelial specific deletion of *Fgfr1* or *Frs2*α encoding FGF receptors inhibited FGF signaling, resulting in enhanced TGF-β signaling and EndMT induction (Chen et al., 2014). Moreover, let-7 miRNA seems to have a crucial function in establishing a bridge between FGF and TGF-β. FGF signaling activation is necessary for the expression of let-7 miRNA, which binds multiple sites on the untranslated region of human *T*β*RI*. Antagonizing FGF signaling diminished the expression of let-7 miRNA, which increased TGF-β1 and TβRI expression and thereby promote TGF-β signaling (Chen et al., 2012). Recently, FGF2 was shown to not only inhibit TGF-β-induced endothelial-to-myofibroblast transition (End-MyoT) mediated via the transcription factor ELK1, but also promoted the formation of active fibroblastic cells with migratory and proliferative characteristics. This revealed the opposing and cooperative action between FGF and TGF-β signaling during the modulation of different mesenchymal cell phenotypes (Akatsu et al., 2019). In mouse embryos with ECs deficient in β-catenin, the cardiac cushion had fewer cells, suggesting that β-catenin in ECs is needed for efficient EndMT and invasion of the mesenchymal cells into the cardiac jelly to form cardiac septa and valves. *In vitro*, TGF-β-induced EndMT was strongly inhibited in β-catenin-knock out ECs, as much less α-SMA was expressed after TGF-β2 stimulation and VE-cadherin levels or Snail1 expression did not change (Liebner et al., 2004). Consistent with this notion, we showed that ECs lacking primary cilia expressed high levels of β-catenin, which was needed to induce Slug expression and subsequent BMP-induced osteogenic differentiation (Sánchez-Duffhues et al., 2015). The Sonic Hedgehog pathway cooperates with TGF-β signaling to stimulate fibroblast differentiation (Horn et al., 2012). Furthermore, inflammatory interleukin (IL)-1β and TGF-β synergistically induce EndMT in HUVECs (Maleszewska et al., 2013). Liguori et al. (2019) showed that the IL-1β/TGF-β2-induced EndMT in HUVECs could be reduced by conditioned medium of adipose derived stromal cells. Katsura et al. (2016) demonstrated that TGF-β signaling engages in crosstalk with the tumor necrosis factor (TNF)-α pathway to enhance EndMT by inducing more miR-31 as a molecular hub, which is required for induction of EndMT. TGF-β suppresses VAV3 and Stk40, which are a negative regulator of MRTF-A (involved in induction of EndMT related gene *ACTA2*) and a suppressor of NF-κB pathway, respectively, in a miR-31-dependent manner. Thus, the lack of Stk40 augments the positive function of miR-31 in EndMT (Katsura et al., 2016). Recently, Glaser et al. (2020) demonstrated that TGF-β2 as well as a combination of IL-1β/TGF-β1 or hypoxia increased the expression of the histone demethylase Jumonji domain-containing protein 2B (JMJD2B) in HUVECs. Interestingly, both siRNA-mediated silencing and pharmacological inhibition of JMJD2B greatly reduced TGF-β2-induced EndMT in HUVECs as demonstrated by a deceased SM22α expression, preserved CDH5 expression and reduced endothelial permeability. The critical function of JMJD2B in EndMT was verified *in vivo*; endothelial specific depletion of JMJD2B in mice resulted in substantial fewer EndMT positive cardiac ECs in the heart after experimentally induced myocardial infarction. However, the reduced EndMT only resulted in a modest rescue of cardiac function 2 weeks after infarction (Glaser et al., 2020).

## EndMT-Related Diseases

**FIGURE 3:**
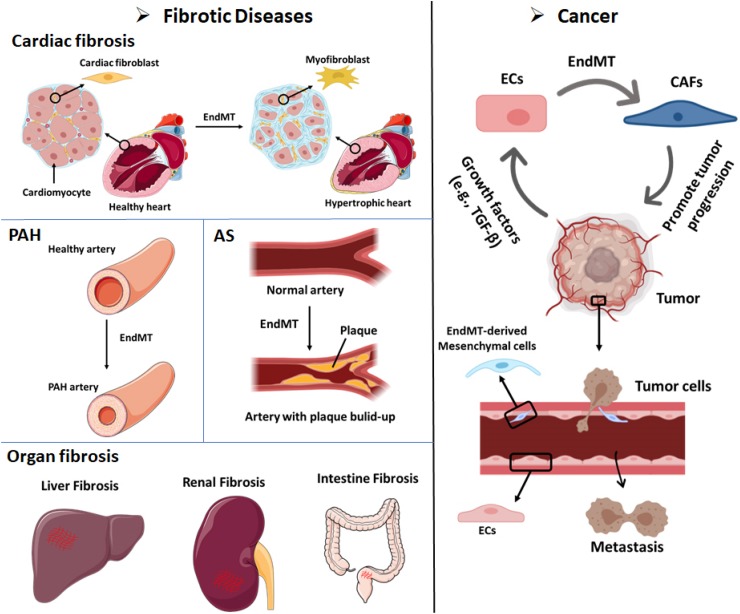
EndMT can contribute to the development of multiple diseases, including fibrotic diseases and cancer. **Cardiac fibrosis:** Endothelial cells that undergo EndMT can differentiate into cardiac fibroblasts that enable cardiac fibrosis. **Pulmonary arterial hypertension (PAH):** The accumulation of EndMT-derived SMA-overexpressing fibrosis cells can thicken and narrow the arterial walls and favor the development of PAH. **Atherosclerosis (AS):** The accumulation of EndMT-derived fibroblasts can lead to plaque growth and facilitate the thickening of AS plaques. **Organ fibrosis:** EndMT-induced fibroblasts have been demonstrated as a source of fibroblast-like cells in liver fibrosis, renal fibrosis, and intestine fibrosis. **Cancer:** (i) Up to 40% of cancer-associated fibroblasts (CAFs) in pancreatic cancer or melanoma animal models were shown to be derived from EndMT. Tumor cells secrete abundant growth factors, including TGF-β, which stimulates endothelial cells to differentiate into CAFs. CAFs can promote cancer invasion and metastasis and immune evasion. (ii) Cancer cell-secreted growth factors (e.g., TGF-β) induce the EndMT of endothelial cells that line tumor blood vessels. EndMT-derived mesenchymal cells weaken the endothelial barrier permeability due to elongation of the cell shape and the loss of adhesion molecules such as claudins and VE-cadherin. These effects facilitate cancer cell intravasation and extravasation.

While EC plasticity and EndMT are important for proper embryonic development, preserving the function of ECs during adult life is an active process and crucial for tissue homeostasis. Endothelial dysfunction can be the consequence of EndMT and can lead to pathological tissue remodeling, thereby contributing to the progression of a variety of diseases, such as fibrotic disorders and tumor development (Figure 3).

### EndMT in Fibrotic Diseases

Fibrotic disorders are characterized by the excessive deposition of matrix produced by an increased number of activated fibroblasts and/or myofibroblasts, which eventually leads to organ dysfunction and systemic disease (Rosenbloom et al., 2017). Although the contribution of ECs to fibrosis is still debatable, results obtained in the past years suggest that EndMT provides an additional source of fibroblasts in fibrotic organs (Zeisberg et al., 2007b, 2008; Piera-Velazquez and Jimenez, 2019). The origin and composition of these fibrosis associated fibroblasts may vary depending on the affected organs. Due to the lack of effective and safe therapies that do not compromise physiological healing, fibrotic diseases constitute a serious health problem and contribute to high mortality. Therefore, there is an urgent need to gain a deeper understanding of the mechanism underlying fibrotic disease to provide the basis for the development of potential antifibrotic treatments, perhaps through the modulation of EndMT.

### Cardiac Fibrosis

Fibrosis in the heart, the accumulation of excessive ECM in the myocardial and perivascular tissues, is an important determinant in the pathogenesis of cardiovascular disorders. Cardiac fibrosis is a response of the heart to stress and injury. Interstitial fibrosis is characterized by unbalanced turnover and excessive deposition of diffuse collagen in the interstitial space and it is often found under conditions of pressure and/or volume overload, in metabolic disorders, or following ischemic insults (Frangogiannis, 2019). Replacement fibrosis mainly occurs after myocardial infarction in the healing ventricle, where dead myocardial cells are substituted by a collagen-based fibrotic scar (Prabhu and Frangogiannis, 2016). Cardiac fibrosis compromises the contractile function of the heart, leading to impaired ventricular relaxation and eventually ventricular hypertrophy, reduced cardiac output, and heart failure (Mocumbi et al., 2019). Whether EndMT contributes to the pool of cardiac fibroblasts remains controversial and depends on the affected tissue. Using a *Tie1Cre;R26RstoplacZ* fate mapping strategy, Zeisberg et al. (2007b) showed an increase in LacZ-positive cells that co-expressed the fibroblast marker FSP1 surrounding the cardiac capillaries. Furthermore, the authors demonstrated how activated Smad2/3 was increased in these cells, and that the knockout of *Smad3* decreased EndMT and reduced cardiac fibrosis (Zeisberg et al., 2007b). Notably, neither Tie1 nor FSP1 are exclusively expressed in ECs or fibroblasts, respectively (Van Amerongen et al., 2008; Kong et al., 2013). Furthermore, whether the labeled Tie1^+^ fibroblasts are derived from cardiac ECs or whether they are derived from existing fibroblasts that originated during cardiac development, and proliferated in response to tissue damage, remains unknown. Therefore, additional studies using alternative endothelial and fibroblast markers and/or inducible (postnatal) reporter strategies are needed.

### Pulmonary Arterial Hypertension

Pulmonary arterial hypertension (PAH) is a disease characterized by progressive thickening and narrowing of the pulmonary arterial walls (Farber and Loscalzo, 2004). This leads to increased resistance in the pulmonary circulation, which negatively impacts the cardiac left ventricle that becomes hypertrophic (Farber and Loscalzo, 2004). Inactivating gene mutations affecting the *BMPRII* have been found in 70% of familial PAH cases and in 10–40% of idiopathic PAH cases (Ranchoux et al., 2015). Moreover, non-genetic cases of PAH exhibit decreased expression of *BMPRII* (Orriols et al., 2017), likely due to an inflammatory environment that negatively affects the expression of BMPRII (Hurst et al., 2017; Sánchez-Duffhues et al., 2019a). Using two different endothelial reporter mice (i.e., Tie2 and VE-cadherin) in combination with immunostaining for α-SMA and MYH11, Qiao et al. (2014) demonstrated the occurrence of EndMT in pulmonary vessels in an experimental animal model of PAH induced by monocrotaline and pneumonectomy. More recent studies combining immunofluorescent labeling and confocal imaging confirmed the presence of EndMT in lung sections from PAH patients (Ranchoux et al., 2015). Furthermore, Good et al. (2015) demonstrated the presence of transitional EndMT cells in the lungs of both hypoxia/SU5416 mice (a murine PAH model) and PAH patient samples by the colocalization of vWF and α-SMA expression. More EndMT cells (vWF and α-SMA double-positive cells) were found in hypoxia/SU5416 mice sections and patient samples. Pulmonary artery ECs (PAECs) undergo EndMT following stimulation with the inflammatory cytokines IL-1β, TNFα, and TGF-β, and in turn secrete more proinflammatory cytokines that may further promote PAH progression (Good et al., 2015). Hopper et al. (2016) showed that dysfunctional BMPRII signaling in PAECs upregulated the expression of High Mobility Group AT-hook 1 (HMGA1), which might promote EndMT and contribute to PAH. Zhao et al. (2020) found that overexpression of miR-181b in the lung inhibited the monocrotaline-induced PAH-like phenotypic response in rats as demonstrated by a decreased right ventricular systolic pressure (RVSP), mean pulmonary artery pressure (mPAP), pulmonary vascular hypertrophy, and right ventricular remodeling. Mechanistically, overexpression of miR-181b in rat pulmonary arterial ECs (rPAECs) was found to inhibit TNF-α, TGF-β1, and IL-1β-induced EndMT by inhibiting the expression of TGF-βR1 and circulating proteoglycan endocan (Zhao et al., 2020).

### Atherosclerosis

Atherosclerosis (AS) refers to the formation of atherosclerotic, calcified plaques. Although still asymptomatic, the vascular remodeling associated to this progressive condition is thought to begin after the first decade of life, due to the combined action of cytokines that induce the accumulation of SMCs, fibroblasts, and osteoblasts in the arterial wall, resembling the process of endochondral bone formation (Souilhol et al., 2018; Kovacic et al., 2019). The expansion and rupture of atherosclerotic plaques may disturb the blood flow and lead to myocardial infarction, stroke, aneurysm, or pulmonary embolism (Alexopoulos and Raggi, 2009). Although many different groups (including ours) have identified ECs as a source of mesenchymal cells within the plaque, two groups have confirmed the presence of double-positive endothelial–mesenchymal cell populations using lineage tracing strategies (Chen et al., 2015; Evrard et al., 2016). As such, Evrard et al. (2016) made use of the tamoxifen-inducible endothelial-specific lineage tracing system end*SclCreERT;R26RstopYfp* in a pro-atherosclerotic *ApoE^–/–^* background to identify double-positive FSP-1/vWF or fibroblast activating protein (FAP)/CD31 cells in vulnerable atherosclerotic lesions. By using *in vitro* modeling, they found that both oxidative stress and hypoxia, which are hallmarks of AS, enhanced TGF-β-induced EndMT (Evrard et al., 2016). In an elegant study by Chen et al. (2015), using VE-cadherin-labeled reporter mice in combination with an *ApoE^–/–^ Frs2a*^*ECKO*^ atherogenic background, increased TGF-β signaling was observed to be related to EndMT in atherosclerotic plaques. Kim et al. (2013) showed that AS might be a severe side effect of radiation by inducing EndMT. Radiation can induce EndMT in heart aortic ECs (HAoECs), accompanied by the decreased expression of CD31 and VE-cadherin and increased expression of FSP-1 and α-SMA. They observed more atherosclerotic plaques in irradiated than in non-irradiated *ApoE^–/–^* mice. By immunofluorescence staining of aortic sinus sections for endothelial CD31 and mesenchymal α-SMA marker proteins, higher levels of cells undergoing EndMT were found in the irradiated *ApoE^–/–^* mice, which suggests that radiation-triggered EndMT might promote AS (Kim et al., 2013).

### Organ Fibrosis

EndMT has also been implicated in the development of fibrosis in other organs, such as the lung, kidney, and liver (Piera-Velazquez et al., 2016). The origin of the fibroblasts in kidney fibrosis was studied by Zeisberg et al. (2008) using three different mouse chronic kidney disease models. In the kidney sections, up to 50% of fibroblasts showed the expression of both an endothelial marker (CD31) and fibroblast and myofibroblast markers (FSP-1 and α-SMA, respectively). Their results suggest the contribution of EndMT to the accumulation of fibroblasts in the kidney and related renal fibrosis diseases. Li et al. (2009) also provided evidence that EndMT occurs and promotes the early development of diabetic renal interstitial fibrosis. They used endothelial lineage tracing with Tie2-cre;LoxP-enhanced green fluorescent protein (EGFP) mice to distinguish endothelial-derived cells. A considerable number of ECs in the fibrotic kidneys of diabetic nephropathy mice were found to express α-SMA. α-SMA positive cells with an endothelial origin were also found in afferent/efferent arterioles in glomeruli, suggesting that the EndMT-derived myofibroblasts can promote glomerulosclerosis (Li et al., 2009). However, in the literature and at scientific meetings discussion remains about existence of EndMT (and EMT) in kidney fibrosis (Cruz-Solbes and Youker, 2017). EndMT has also been linked to liver fibrosis. The liver tissue sections from idiopathic portal hypertension (IPH) showed double-positive staining for CD34 and S100A4, which are EC and myofibroblast markers, respectively. Based on an increase in phosphorylated Smad2 levels, TGF-β signaling may be linked to EndMT in the portal vein endothelium and lead to eventual portal vein stenosis and obliteration in IPH (Kitao et al., 2009). A recent report showed that defective autophagy induced by suppression of *ATG5* expression resulted in EndMT in human microvascular ECs (HMVECs) mediated by an abnormal accumulation of IL-6. Feeding endothelial-specific *ATG5* knockout mice with high-fat diet (HFD) resulted in profound tubular damage and interstitial fibrosis in the kidney and stronger perivascular fibrosis in the heart compared to control animals. Increased EndMT was also found in *ATG5* deficient mice, which supported the notion that disruption of autophagy triggers EndMT can contribute to organ fibrosis *in vivo* (Takagaki et al., 2020).

### EndMT in Cancer

ECs and angiogenesis are known to have critical function in tumor development and metastasis (Sobierajska et al., 2020). Emerging evidence has shown that EndMT not only plays roles in promoting cancer development and metastasis, but also influences the response to cancer therapy (Potenta et al., 2008; Platel et al., 2019). Tumor progression is facilitated by fibroblasts within the tumor. The origin of these CAFs has been investigated using Tie1Cre;R26R stop lacZ transgenic mice, and up to 40% of the CAFs in pancreatic cancer or melanoma models may have originated from EndMT (Zeisberg et al., 2007a). CAFs facilitate cancer progression by influencing the tumor microenvironment. CAFs secrete various cytokines and chemokines that influence the behavior of different cell types (Allen and Louise Jones, 2011; Polanska and Orimo, 2013). For example, VE growth factor (VEGF), which is secreted by CAFs, promotes vascular formation at tumor sites and may thereby provide more nutrition for tumor growth. CAFs secrete TGF-β to promote cancer invasion and metastasis (Xiao et al., 2015). Other CAF-derived factors, such as EGF, FGF, and matrix metalloproteinases (MMPs), have been identified as contributors of cancer progression that promote proliferation and invasion (Mendelsohn and Baselga, 2000; Katoh and Nakagama, 2014; Ciszewski et al., 2017). Interestingly, CAFs may also play a role in awakening dormant cells to induce metastasis (De Wever et al., 2014). In addition to supporting the fibroblast population, EndMT may contribute to weakening of the endothelial barrier due to the elongation of the cell shape and the loss of adhesion molecules such as claudins and VE-cadherin, supporting tumor metastasis (Anderberg et al., 2013; Gasparics et al., 2016). Krizbai et al. (2015) found that after inducing EndMT by treating ECs with cancer cell conditioned medium, the transendothelial electrical resistance was decreased indicative for loss of barrier function, and more melanoma cells were able to adhere to ECs and transmigrated through the endothelial layer. Therefore, EndMT might play a role during metastatic trans-endothelial migration.

Moreover, recent studies showed that the response of cancer cells to chemo- and targeted therapy can be influenced by EndMT. Kim et al. (2019) showed that HUVECs undergoing EndMT enhanced the resistance of tumor spheroids against EGFR inhibitor gefitinib and chemotherapy cisplatin. Furthermore, CAFs originated at tumor sites via EndMT influence chemotherapy in several ways. CAFs secrete some factors, such as IL-6 and IL-8, and matricellular proteins to regulate chemoresistance (Shintani et al., 2016; Leask, 2019). At the same time, CAFs reduce the levels of therapeutic reagents in tumors by decreasing the expression of drug transporters and trapping active agents (Chen and Song, 2019). EndMT is also related to radiation therapy. Choi et al. (2018) showed that radiation could induce EndMT, which triggered tumor-associated macrophage (TAM) polarization toward an M2 phenotype and resulted in radiation resistance. Additionally, CAFs can support immune evasion and act as an immunosuppressive agent in cancer immunotherapy, by inducing the secretion of multiple chemokines and cytokines, such as TGF-β and IL-6/8/13, and thereby inhibit the antitumor immune response. Additionally, the ECM produced by CAFs at tumor sites enhances ECM stiffness and obstructs the infiltration of effector T cells into the tumor (Chakravarthy et al., 2018; Liu et al., 2019; Monteran and Erez, 2019). In conclusion, EndMT is a promising target for cancer therapy, although more investigation is needed.

### EndMT in Cerebral Cavernous Malformation

EndMT has also been shown to contribute to the development of CCM, a disease that can result in brain hemorrhage, seizure, and paralysis (Bravi et al., 2015, 2016). Loss-of-function mutations in CCM1 is one of the causes of CCM. In endothelial-specific *Ccm1* (also known as KRIT1)-ablated mice, ECs in the vascular lesions of the brain underwent EndMT; N-cadherin was increased that promoted the formation of vascular malformations. The deletion of *Ccm1* in ECs upregulated the secretion of BMP6 and, in turn, increased the sensitivity of the response to TGF-β and activated BMP signaling to induce EndMT (Maddaluno et al., 2013). EndMT was shown to be critical in the onset and progression of CCM. In line with these results, Takada et al. (2017) found that ECs in cerebral and orbital CCM expressed both the endothelial marker CD31 and the mesenchymal markers α-SMA and CD44, also demonstrating the occurrence of EndMT.

## EndMT in Tissue Regeneration and Engineering

**FIGURE 4:**
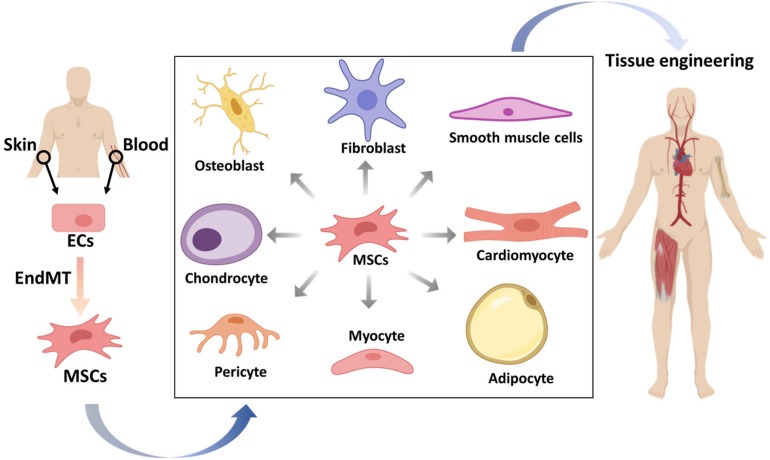
The potential applications of mesenchymal stem cells (MSCs) that originate from endothelial cells (ECs) in tissue engineering. ECs from patients isolated from tissues, such as skin or blood, can be stimulated to undergo endothelial to mesenchymal transition (EndMT) to generate MSCs. These multipotent MSCs can be differentiated into various cell types, which may be used to form desired tissue types that can be transplanted into patients.

In addition to the pathological effects of (myo)fibroblast generation, the beneficial aspects of EndMT are gradually being discovered. EndMT has the potential to drive ECs to mesenchymal multipotent cells (MSCs), able to further differentiate into various different cell types that can be applied in tissue engineering and regeneration (Medici and Kalluri, 2012; Figure 4).

The ability of EndMT to generate various cell types has been described *in vivo* and *in vitro*. Fibrodysplasia ossificans progressiva (FOP) patients, which suffer from heterotopic bone formation, have a gain-of-function mutation in the BMP type I receptor ALK2 (Shore et al., 2006). Endothelial-like cells were identified as a source of heterotopic cartilage and bone formation in Tie2-GFP reporter mice injected with adenoviral particles expressing a constitutively active form of ALK2. Moreover, immunostaining performed in patient-derived tissue sections revealed the existence of double positive cells expressing either Tie2 or vWF and Osteocalcin (osteoblast marker) or SOX9 (chondrocyte marker). Overexpression of this mutated ALK2 in ECs induced EndMT, and the cells adapted the characteristics of MSCs, which have the ability to differentiate into osteoblasts, chondrocytes, or adipocytes. Similar results were found in TGF-β-treated cells, which verified the utilization of TGF-β-induced EndMT to generate MSCs (Medici et al., 2010). Although whether ECs contribute to ectopic bone formation in FOP patients remains controversial, we have recently demonstrated how circulating ECs isolated from FOP donors exhibit enhanced EndMT and osteogenic differentiation *in vitro*, which was used as a functional readout to identify novel small molecules targeting ALK2 (Sánchez-Duffhues et al., 2019b). This illustrates the potential of EndMT to establish surrogate models for research without the need to go through iPSCs. Osteoprogenitor cells formed after the EndMT process were also found in calcifications of the aortic tract (Yao et al., 2015; Boström et al., 2016), valves (Hjortnaes et al., 2015), and tumors (Dudley et al., 2008). Furthermore, via VE-cadherin lineage tracing in mice, EndMT was also shown to be involved in the transformation of ECs into white and brown fat cells (Tran et al., 2012). Recent manuscripts identified that microvascular ECs within adipose tissue in patients with obesity undergo EndMT, thereby modifying their secretome and enhancing systemic inflammation (Haynes et al., 2019). ECs isolated from tumor vessels can undergo EndMT to subsequently differentiate into adipocytes, pericytes, and SMCs (Huang et al., 2015), which suggests that artificially modified EndMT-derived cells may be useful to induce tissue repair in a paracrine manner. Furthermore, ECs were discovered to have the potential to form skeletal myocytes in muscle repair (Huang et al., 2014). ECs also contribute to cardiac renewal (Fioret et al., 2014). Evidence has also shown that a subset of valvular ECs behave as progenitor cells that can undergo EndMT and replenish valvular interstitial cells to repair valves (Bischoff and Aikawa, 2011).

The potential of ECs to generate different cell types via EndMT makes steering this process a potential tool in tissue regeneration. For example, EndMT-derived osteoblasts or chondrocytes could be used in skeletal conditions, such as osteoporosis, bone fracture healing, or osteoarthritis. In addition, EndMT-induced myogenesis may generate cardiomyocytes to alleviate myocardial infarction (Medici, 2016). EndMT-mediated chondrogenesis could be employed in osteoarthritis or temporal mandibular joint disorder (TMJD) therapies. Due to its ability to generate SMCs and pericytes, steering EndMT could be an option for vascular formation-related tissue engineering. EndMT might also have the potential to promote angiogenesis as Snail1 mediated EndMT was shown to play a role in regulating vessel formation (Sun et al., 2018). Zheng et al. (2007) showed that myoendothelial cells isolated from human skeletal muscle have the potential to differentiate into myogenic, osteogenic, and chondrogenic cells after culturing in special formulated media supplemented with cytokines. After injecting isolated human myoendothelial cells into damaged muscles in immune compromised mice, dystrophin and human-specific lamin A/C double positive myofibers were observed in mice muscle. This result suggests the potential of regulating myoendothelial cells differentiation for the treatment of muscle related disease.

The potential of EndMT may also be considered in combination with the emerging use of organ-on-chips. ECs grown *in vitro* on chips can mimic the function of blood vessel networks, e.g., they contain a functional endothelial lumen sensitive to flow. Moya et al. (2013) set up a 3D dynamic perfused capillary network model *in vitro* using human endothelial colony forming cell-derived ECs (ECFC-ECs) isolated from cord blood. In addition, Mathur et al. (2019) explored the potential of blood outgrowth ECs (BOECs), which were isolated from venous circulation, to reconstitute vascular networks on vessel-chips. The authors used this 3D complex model constituted with swine BOECs to study the response of the endothelium in diabetes. Noteworthy, perfusion of 3D vessels with whole blood from diabetic pigs led to an enhanced formation of thrombi compared to control animals, such as lower proliferation, more intact lumen, reactive oxidative stress, and platelet adhesion, which also are expected in diabetic patients. This demonstrates the possibility of developing personalized vessel structures on a chip device (Mathur et al., 2019). Although EndMT was not the specific aim of the study, Kolesky et al. (2014) successfully developed a 3D chip resembling vascular calcification using a bio-printing approach with three different cell types (i.e., mesenchymal stem cells, fibroblasts, and ECs). This perfusable vascular tissue was useful to study vascular calcification and monitor osteocalcin expression and collagen deposition.

*In vitro* 3D organ cultures can be used to study EndMT-related diseases. For example, Wagner et al. (2020) established 3D vascularized cardiac tissue mimetics (CTMs) by co-culturing cardiomyocytes (CM) and fibroblasts (FB) in spheroids and then complementing them with HUVECs to investigate the heterocellular crosstalk in different culture conditions. In this system, TGF-β stimulation could induce EndMT as vimentin/SM22α was expressed in Isolectin B4 stained ECs, and more vascularization was observed in CTMs. In summary, although not so many mature applications have been established to date, the role of TGF-β-induced EndMT in tissue engineering and 3D *in vitro* modeling is emerging.

## Conclusion

EndMT, a complex process in which ECs change their morphology into that of fibroblast-like mesenchymal cells, is accompanied by changes in cell function and endothelial and mesenchymal marker protein expression. TGF-β, a major inducer of EndMT, regulates the underlying mechanisms via the Smad/non-Smad signaling pathways and interacts with other signaling cascades to orchestrate this process. An in-depth understanding of the dynamic mechanisms of TGF-β signaling in the EndMT process would help to precisely regulate this transition. The EndMT process is a double-edged sword. EndMT is needed for proper development of the embryo and wound healing, but also contributes to some fatal diseases, such as tissue fibrosis and cancer. Inhibition of the EndMT process, e.g., by inhibiting TGF-β signaling, is being pursued for the treatment of diseases associated with/caused by EndMT. But, the discovery of EndMT-derived multipotent cells has inspired scientists to explore the therapeutic potential of TGF-β-induced EndMT in tissue regeneration and tissue engineering. Since almost all tissues in the body are highly vascularized, the EndMT-derived multipotent cells in vascular engineering might be applied in other cell types to enable the regeneration of a well-contained vascular tissue. In addition, resident ECs within or near damaged tissues could be used in a similar way to enable tissue repair by reprogramming them into mesenchymal multipotent cells and thereafter stimulating the formation of differentiated derivatives. The potential of EndMT in tissue regeneration and engineering is promising.

## Author Contributions

JM wrote the initial draft of the manuscript. GS-D and M-JG provided feedback and comments on the manuscript. PD supervised and coordinated the writing. All authors have approved the manuscript for publication.

## Conflict of Interest

The authors declare that the research was conducted in the absence of any commercial or financial relationships that could be construed as a potential conflict of interest.
